# Redirecting Valvular Myofibroblasts into Dormant Fibroblasts through Light-mediated Reduction in Substrate Modulus

**DOI:** 10.1371/journal.pone.0039969

**Published:** 2012-07-13

**Authors:** Huan Wang, Sarah M. Haeger, April M. Kloxin, Leslie A. Leinwand, Kristi S. Anseth

**Affiliations:** 1 Department of Molecular, Cellular and Developmental Biology, University of Colorado, Boulder, Colorado, United States of America; 2 Department of Chemical and Biological Engineering, University of Colorado, Boulder, Colorado, United States of America; 3 Department of Chemical and Biomolecular Engineering and Department of Materials Science and Engineering, University of Delaware, Newark, Delaware, United States of America; 4 Howard Hughes Medical Institute, University of Colorado, Boulder, Colorado, United States of America; 5 Biofrontiers Institute, University of Colorado, Boulder, Colorado, United States of America; Brigham and Women's Hospital, Harvard Medical School, United States of America

## Abstract

Fibroblasts residing in connective tissues throughout the body are responsible for extracellular matrix (ECM) homeostasis and repair. In response to tissue damage, they activate to become myofibroblasts, which have organized contractile cytoskeletons and produce a myriad of proteins for ECM remodeling. However, persistence of myofibroblasts can lead to fibrosis with excessive collagen deposition and tissue stiffening. Thus, understanding which signals regulate de-activation of myofibroblasts during normal tissue repair is critical. Substrate modulus has recently been shown to regulate fibrogenic properties, proliferation and apoptosis of fibroblasts isolated from different organs. However, few studies track the cellular responses of fibroblasts to dynamic changes in the microenvironmental modulus. Here, we utilized a light-responsive hydrogel system to probe the fate of valvular myofibroblasts when the Young’s modulus of the substrate was reduced from ∼32 kPa, mimicking pre-calcified diseased tissue, to ∼7 kPa, mimicking healthy cardiac valve fibrosa. After softening the substrata, valvular myofibroblasts de-activated with decreases in α-smooth muscle actin (α-SMA) stress fibers and proliferation, indicating a dormant fibroblast state. Gene signatures of myofibroblasts (including α-SMA and connective tissue growth factor (CTGF)) were significantly down-regulated to fibroblast levels within 6 hours of *in situ* substrate elasticity reduction while a general fibroblast gene vimentin was not changed. Additionally, the de-activated fibroblasts were in a reversible state and could be re-activated to enter cell cycle by growth stimulation and to express fibrogenic genes, such as CTGF, collagen 1A1 and fibronectin 1, in response to TGF-β1. Our data suggest that lowering substrate modulus can serve as a cue to down-regulate the valvular myofibroblast phenotype resulting in a predominantly quiescent fibroblast population. These results provide insight in designing hydrogel substrates with physiologically relevant stiffness to dynamically redirect cell fate *in vitro.*

## Introduction

The microenvironment of a cell regulates cellular functions dynamically [1], [2], [3]. In particular, substrate elasticity has recently been shown to direct cell functions, such as stem cell differentiation [4] and renewal [5], independent of soluble growth factors. Soft matrices that mimic the stiffness of brain with Young’s modulus (*E*) around 0.1–1 kPa promote neurogenic differentiation of human mesenchymal stem cells, while rigid matrices that mimic collagenous bone (*E,* 25–40 kPa) promote osteogenesis [4]. Further, rigid plastic tissue culture plates have a non-physiological stiffness (10,000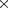 that of soft tissue) and are known to affect cell phenotypes. For example, when fibroblasts are cultured on plastic plates, they spontaneously differentiate into myofibroblasts with fibrogenic properties [6], [7], [8]. In contrast, soft substrata derived from polyacrylamide- or poly(ethylene glycol)-based hydrogels with physiologically-relevant moduli (*E*10kPa) inhibit myofibroblast differentiation, better preserving the inactivated cellular phenotype [9], [10], [11]. From this perspective, hydrogels with a tissue-mimicking elastic modulus provide an important culture system to study and direct fibroblast functions *in vitro*.

When fibroblasts are transformed into myofibroblasts over long time periods *in vivo*, tissue fibrosis can develop [12], [13]. Tissue fibrosis presents serious health problems affecting multiple organs, including skin [14], lung [15], liver [16], kidney [17] and heart [18]. Resident fibroblasts in these tissues have been shown to play critical roles in disease progression. Fibroblasts respond to both chemical cues and the physical stiffness of tissue to become myofibroblasts [18], [19], [20]. For example, transforming growth factor β1 (TGF-β1) is a potent profibrotic cytokine that activates fibroblasts from valve, skin or liver to become myofibroblasts [9], [18], [21]. Myofibroblasts, characterized by increased secretion of ECM proteins (e.g., collagen and fibronectin) and higher contractile function mediated through α-smooth muscle actin (α-SMA) stress fibers, exacerbate fibrosis [20], [22]. Strategies to reverse the myofibroblast phenotype into a native fibroblastic phenotype could be of significant therapeutic impact in abrogating tissue fibrosis.

Primary fibroblasts isolated from pig aortic valves serve as a model system to study how the pathogenic myofibroblast phenotype is regulated by microenvironment modulus [23], [24], [25]. These fibroblasts, valvular interstitial cells (VICs), are the main cell population residing in aortic valves [26]. In a healthy valve, VICs maintain a quiescent fibroblastic phenotype; however, in a sclerotic valve, VICs are activated to myofibroblasts, which secrete excessive ECM degradative enzymes (e.g., MMPs) and collagen, leading to deterioration of the original valve structure and tissue thickening [27], [28], [29]. Persistence of the myofibroblast phenotype leads to further valve stiffening, which eventually restricts blood flow from the left ventricle to the aorta. Increased rigidity of valvular tissue associated with aortic valve (AV) sclerosis is not only a result of collagen deposition by myofibroblasts, but also can promote pathology by a positive feedback mechanism, leading to the accumulation of myofibroblasts and the continual production of ECM [30].

Microenvironment stiffness has been shown to regulate the fate of fibroblasts, including differentiation into myofibroblasts, apoptosis and proliferation. When valvular, hepatic or lung fibroblasts are cultured on low modulus substrata (*E
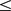
*10 kPa), they maintain an un-activated phenotype; however, when cultured on higher modulus substrata, they are activated to myofibroblasts [10], [11], [31], [32]. Reducing the elastic modulus of the substratum to a very low level (*E
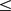
*1 kPa) promotes apoptosis in various fibroblasts [11], [33], [34]. Moreover, NIH3T3 fibroblasts grown on compliant substrata (*E* ∼ 5 kPa) show a decrease in proliferation, compared with cells cultured on stiff substrata (*E* ∼ 14 kPa) [33]. Based on previous findings, we speculated that substrata with *E* lower than 10 kPa, but not too low to activate apoptosis, may provide healthy physical signals to maintain quiescent fibroblast phenotypes. Specifically in this study, we test the hypothesis that the valvular myofibroblasts will either undergo apoptosis or de-activate to a quiescent fibroblast state when the substrate modulus is reduced.

We utilized a photodegradable poly(ethylene glycol) (PD-PEG) hydrogel [35] to study the fate of VICs in response to substrate modulus reduction. With this unique photo-sensitive material, we can change *E in situ* while VICs are adhered to the gels from ∼32 kPa, mimicking collagenous bone (which has been detected in diseased valves [36]), to ∼7 kPa, mimicking healthy valve fibrosa [31]. We found that VICs switched their fate from activated myofibroblasts to fibroblasts with reduced proliferation when the substrate was changed from stiff to soft. This de-activation process was not associated with significant apoptosis, but was characterized by down-regulation of critical myofibroblast phenotypic markers, including loss of α-SMA stress fibers, significant reduction in the expression of myofibroblast gene signatures (α-SMA and CTGF) and a significant decrease in proliferation. These results provide insight into the potential fate of valvular myofibroblasts *in vivo* after tissue repair. Further, we established that de-activated VICs still maintain the potential to activate the expression of myofibroblast genes in response to TGF-β1 and to proliferate in response to growth stimuli, indicating a reversible fibroblast state of the cells and contributing to our understanding of how modulus regulates the myofibroblast-fibroblast transition. This could be useful in developing novel treatments for tissue fibrosis and could result in new approaches to direct cell fate and function for tissue engineering.

## Materials and Methods

### Hydrogel Synthesis

The photodegradable crosslinker (PD-PEG) was synthesized as previously described [35]. PD-PEG (M_n_ ∼4070 g/mol, 8.2 wt%) was copolymerized with PEG monoacrylate (PEGA, M_n_ ∼400 g/mol, 6.8 wt%, Monomer-Polymer and Dajac Laboratories, Inc) and an acrylated adhesion peptide sequence RGDS (5 mM, described below) via redox-initiated free radical chain polymerization [37]. The hydrogels were synthesized as thin films (0.25 mm thick) covalently attached to methacrylated coverglass. Hydrogels with surface areas (A) of ∼ 250 mm^2^ and ∼ 370 mm^2^ were made on coverglass of 18 mm and 22 mm in diameter, respectively, to enable harvest of appropriate cell numbers for specific assays. To reduce the crosslinking density and modulus of the hydrogels, samples were irradiated with long wavelength, low intensity ultraviolet (UV) light for 5 minutes (365 nm at 10 mW/cm^2^). These conditions have been previously demonstrated to be cytocompatible [10]. Hydrogel moduli were verified with rheometry and atomic force microscopy as described previously [10], where moduli of hydrogels (0.25 mm thick) in phosphate buffered saline (PBS) were measured as *E* ∼32 kPa and ∼7 kPa after 0 minute and 5 minutes of irradiation (365 nm at 10 mW/cm^2^), respectively.

### Peptide Synthesis

An integrin-binding adhesion peptide was synthesized and incorporated within the cell culture platform to promote cell attachment based on established protocols [37], [38] Briefly, OOGRGDSG (diethylene glycol-diethylene glycol-glycine-arginine-glycine-aspartic acid-serine-glycine) was made on a solid phase peptide synthesizer (Tribute, Protein Technologies, Inc.) with HBTU/HOBt amino acid activation and Fmoc chemistry [38]. After synthesis of the primary sequence, the N-terminus of the peptide was modified on resin with an acryloyl group by reaction with HATU-activated acrylic acid in the presence of diisopropylethylamine (4 molar excess of each relative to N-terminus amine) [39]. Complete reaction of the amine was verified using the Kaiser test [40], and the peptide was cleaved from resin (5 wt% phenol in 95% trifluoroacetic acid, 2.5% triisopropylsilane, and 2.5% DI water) stirring for 2 hours at room temperature. The cleavage solution was precipitated in and washed with ice cold ethyl ether (3x), dried under vacuum overnight, purified by high-performance liquid chromatography (HPLC), and characterized by matrix-assisted laser desorption/ionization mass (MALDI-MS) [37]. The peptide has a molecular weight of ∼892 g/mol, consistent with its amino acid sequence.

### Cell Culture

Fresh porcine hearts were obtained from Hormel Foods Corporation (Austin, MN, USA) within 24 hours of sacrifice and aortic valve leaflets were excised. Primary VICs were harvested from porcine aortic valve leaflets based on a sequential collagenase digestion as previously described [41]. The isolated cells were cultured in growth medium (Medium 199, 15% fetal bovine serum (FBS), 50 U/ml penicillin, 50 µg/ml streptomycin, and 0.5 µg/ml fungizone) and expanded up to passage 3. Passage 3 VICs were seeded on hydrogels at 35,000 cells/cm^2^ and were cultured in low serum medium (Medium 199, 1% FBS, 50 U/ml penicillin, 50 µg/ml streptomycin, and 0.5 µg/ml fungizone) for up to 5 days. Treatment with fibroblast growth factor 2 (FGF2, Sigma Cat# F0291, 16 ng/ml) in 15% FBS medium or TGF-β1 (R&D systems, Cat# 101-B1-001, 5 ng/ml) in 1% FBS medium was applied on day 4 for 24 hours.

### Immunocytochemistry

Hydrogels (A ∼ 250 mm^2^) were fixed with 4% paraformaldehyde (overnight at 4°C), permeabilized in 0.1% TritonX100, and blocked with 5% bovine serum albumin (BSA). Mouse anti-α-SMA antibody (Abcam, Cat# ab7817) or rabbit anti-vimentin antibody (Cell Signaling Technology, Cat#5741) was diluted at 1∶100 in PBS with 1% BSA and incubated with the samples overnight at 4°C. Following washes in PBS with 0.05% Tween 20, samples were labeled with goat-anti-mouse Alexa Fluor 488 secondary antibody (Life technologies (Invitrogen), Cat# A-11001) or goat-anti-rabbit Alexa Fluor 488 secondary antibody (Life technologies (Invitrogen), Cat# A11070), along with DAPI to visualize nuclei. Samples were subsequently imaged on a LSM 710 Laser Scanning Microscope with transmitted light detector for differential interference contrast (DIC) (Carl Zeiss) or inverted epi-fluorescent microscope (Nikon), each with a 20X magnification objective. Images of different fluorescence channels were compiled with Zen or Metamorph software and analyzed by ImageJ for total nuclei number (Analyze Particles function, NIH). Myofibroblasts were counted as cells with α-SMA staining organized into fibrils, and the percentage of myofibroblasts was calculated as (number of myofibroblasts/total number of cells) x 100%. To quantify each sample, 6 random fields of view were imaged, counted, and averaged.

### Annexin V Staining

VICs were washed in PBS and detached from hydrogels (A ∼ 370 mm^2^) using TrypLE (Life technologies, Cat# A1285901). Following centrifugation, cell pellets were re-suspended in Annexin V-binding buffer (10 mM HEPES, 140 mM NaCl, 2.5 mM CaCl_2_, pH 7.4) and incubated with Alexa Fluor 594-labeled Annexin V (Life technologies, Cat# A13203) for 15 minutes at room temperature. DAPI was applied at 0.5 µg/ml to distinguish live and dead cell populations. Percent of Annexin V^+^ cells was quantified using a CyAn ADP flow cytometer (Beckman Coulter). Early apoptotic cells were identified as those with high fluorescent emission in the Alexa Fluor 594 channel and low emission in the DAPI channel. On average, over 10,000 events were collected per sample. As a positive control, VICs were treated for 18 hours with 25 µM camptothecin, an apoptosis-inducing reagent.

### EdU Labeling

For quantifying basal level proliferation amongst different substrate moduli, VICs were switched to medium with 5% FBS after photodegradation on day 3 and were subsequently incubated with 10 µM EdU for 3 hours on day 5. For examining proliferative response to growth stimuli after modulus-driven deactivation, VICs were treated with or without FGF2 (Sigma-Aldrich, Cat# F0291, 16 ng/ml) and 15% FBS on day 4 for 24 hours and then incubated with 10 µM EdU for 1 hour on day 5. For each condition, cells from two PD-PEG gels of A ∼ 370 mm^2^ were harvested by trypsinization. For EdU staining, samples were fixed and permeabilized using the Fixation and Permeabilization kit (Life technologies, Cat# GAS003). Following washes in PBS with 1% BSA, the cells were incubated with Click-iT reaction cocktail prepared from the Click-iT EdU Alexa Fluor 488 kit (Life technologies, Cat# C10337) for 30 minutes at room temperature. DAPI was applied at 5 µg/ml as a measure for DNA content. Samples were analyzed by a CyAn ADP flow cytometer (Beckman Coulter). Over 10,000 events were collected per sample. Proliferative cells were counted based on high fluorescence in the Alexa Fluor 488 channel.

### Quantitative Real-Time PCR

For each condition, two PD-PEG gels (A ∼ 370 mm^2^) with attached VICs were harvested and submerged in TRI Reagent (Sigma, Cat# T9424), rapidly frozen in liquid nitrogen, and stored at -80°C until processing. Samples were homogenized with individual DNase and RNase free pestles. Total RNA was purified according to a modified version of the manufacturer’s protocol for TRI Reagent. Briefly, after chloroform extraction and aqueous phase collection, a second chloroform extraction with equal volume of chloroform to aqueous phase was performed. cDNA was synthesized from total RNA with Superscript III reverse transcriptase (Life technologies, Cat# 18080-051) and random hexamer primers. Gene expression was determined by SYBR Green-based quantitative real-time PCR (qRT-PCR) using gene specific primer sets (Table 1) and an Applied Biosystems 7500 Real-Time PCR machine.

**Table 1:** Gene Primer Sequences.

Gene	Forward Primer Sequence	Reverse Primer Sequence
18S	GCCGCTAGAGGTGAAATTCTT	CTTTCGCTCTGGTCCGTCTT
α-SMA	GCAAACAGGAATACGATGAAGCC	AACACATAGGTAACGAGTCAGAGC
CTGF	CTGGTCCAGACCACAGAGTGG	GCAGAAAGCGTTGTCATTGG
Collagen 1a1	GGGCAAGACAGTGATTGAATACA	GGATGGAGGGAGTTTACAGGAA
Fibronectin 1	GGCATTGATGAAGAACCCTTG	GCCTCCACTATGATGTTGTAGGTG
Vimentin	AGGAAGAGATGGCCCGTCA	CCTGCTCTCTTCTCCTTCCA

### Statistics

Data are presented as mean ± standard error of the mean (SEM). SEM was calculated based on three biological replicates. Each biological replicate was based on an isolation of VICs from 60–90 pooled porcine aortic valves as described in 2.2 Cell Culture. A Student's *t-*test was used to compare data sets and a *p* value less than 0.05 was considered statistically significant.

## Results

**Figure 1:**
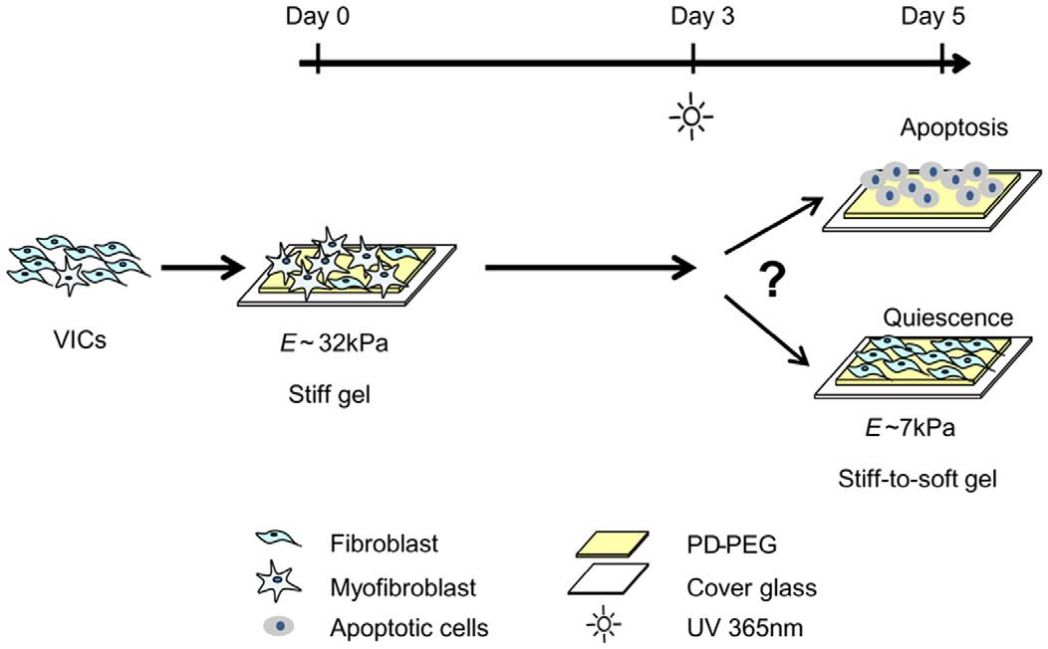
Cell fate in response to substrate modulus reduction. Valvular interstitial cells (VICs) were seeded on photodegradable poly(ethylene glycol) (PD-PEG) gels on day 0. At day 3, a portion of the stiff gels was softened with light (365 nm at 10 mW/cm^2^). The fate of VICs on continuously stiff, continuously soft and stiff-to-soft gels was subsequently examined on day 3 and/or day 5 based on immunocytochemistry, apoptosis staining, proliferative assay and mRNA expression.

We synthesized PD-PEG hydrogels as dynamic culture substrates for VICs [10] with Young’s modulus (*E*) ∼32 kPa (stiff gels), *E* ∼7 kPa (soft gels), and *E* changed from ∼32 kPa to ∼7 kpa (stiff-to-soft gels). A peptide containing the adhesive sequence RGDS was incorporated within the gel to facilitate cell adhesion [42]. VICs were cultured on PD-PEG gels in low serum (1% FBS) medium for up to 5 days (Fig. 1), where the low serum culture limits cellular response (i.e., differentiation or proliferation) to growth factors in the serum [43]. At day 3, half of the stiff gels were irradiated with low intensity UV light to reduce the crosslinking density and modulus (stiff-to-soft gels in Fig. 1). We then investigated the mechanisms of myofibroblast deactivation, examining apoptosis and reversion to quiescence as potential pathways (Fig. 1). Specifically, Annexin V staining was used to examine early apoptosis events. Formation of α-SMA stress fibers, expression of myofibroblast genes, and relative proliferation rate were quantified by immunocytochemistry, qRT-PCR and an EdU-based proliferation assay, respectively.

### Primary Valvular Myofibroblasts Deactivate in Response to Substrate Modulus Reduction

**Figure 2:**
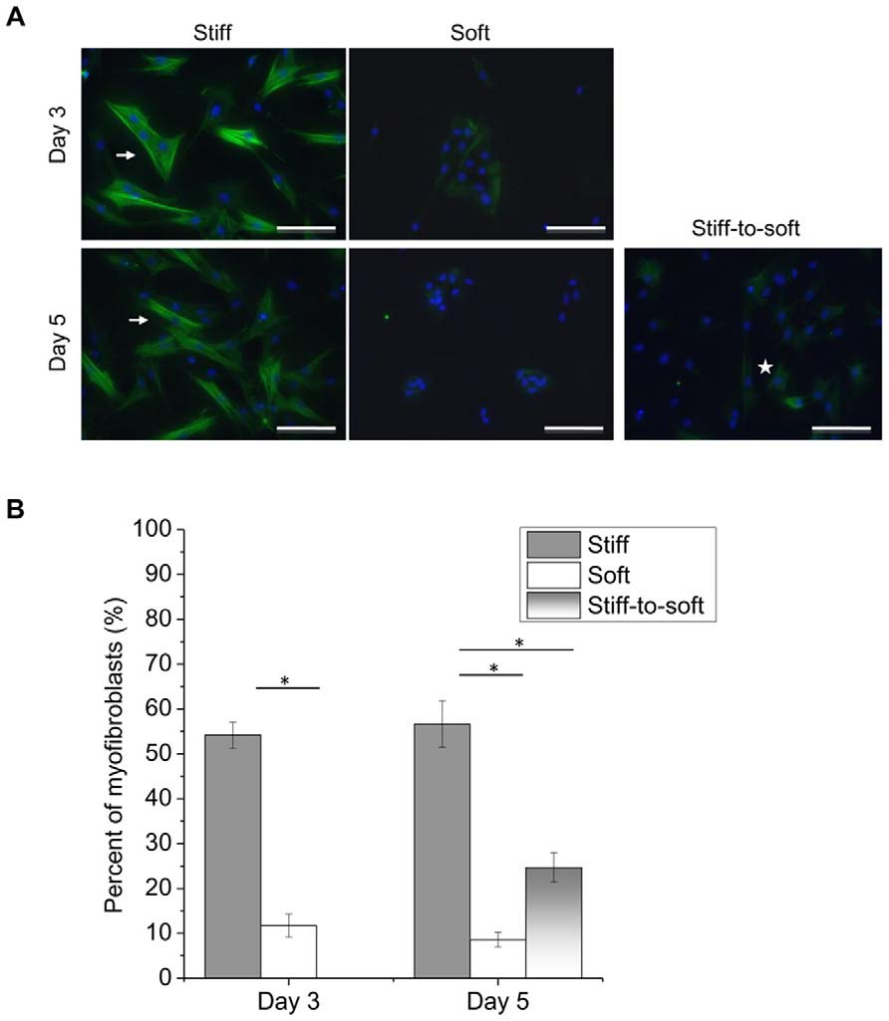
Reduced myofibroblast activation in response to lowering substrate modulus. VICs cultured on stiff, soft or stiff-to-soft gels as shown in Fig.1 were fixed on day 3 and day 5, and stained for α-smooth muscle actin (α-SMA). (A) Representative staining of the myofibroblast phenotype for VICs cultured on substrates with different stiffnesses on day 3 and day 5. Green: α-SMA. Blue: nuclei. Arrows: myofibroblasts characterized by organized α-SMA+ stress fibers. Star: a cell stained with diffusive α-SMA. Scale bar: 100 µm. (B) Quantification of the percent of myofibroblasts on the substrates based on staining in (A). The percentage of myofibroblasts on stiff-to-soft gels or soft gels was significantly lower than that on stiff gels. * indicates p<0.05.

The percent of activated myofibroblasts on different substrates was examined by α-SMA immunocytochemistry (Fig. 2). Fig. 2A shows representative staining of VICs for α-SMA on different gel conditions. Myofibroblasts are defined as cells with α-SMA organized into stress fibers (Fig. 2A, arrows). There were fewer myofibroblasts and lower α-SMA fluorescence intensity on soft and stiff-to-soft gels than on stiff gels (Fig. 2A). Some VICs on softer substrates also showed diffuse α-SMA staining in the cytoplasm (Fig. 2A, star); however, these cells were not classified as myofibroblasts. The percentage of activated myofibroblasts was quantified (Fig. 2B). Stiff gels activated ∼55% of VICs to become myofibroblasts. In contrast, only ∼10% myofibroblasts were observed when VICs were cultured on soft gels (Fig. 2B). From day 3 to day 5, the fraction of myofibroblasts remained at a similar level for both the stiff and the soft gels (Fig. 2B), and the total cell number was not changed significantly on any substrate (Fig. S1). When *E* was reduced from 32 kPa to 7 kPa (stiff-to-soft gels, Fig. 2B), the percentage of myofibroblasts decreased from 56.7
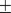
5.2% to 24.7
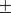
3.2% over the course of 2 days.

### The Decrease in the Proportion of Valvular Myofibroblasts is not due to Apoptosis

**Figure 3:**
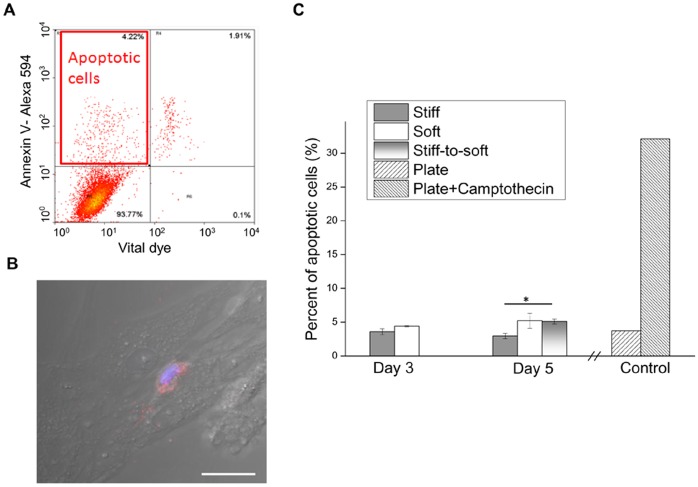
Decreased number of myofibroblasts on stiff-to-soft gels was not due to apoptosis. VICs cultured on different PD-PEG gels were stained with Annexin V linked with Alexa Fluor 594 and DAPI to detect apoptosis. (A) Scatter plot of Annexin V and DAPI staining for VICs cultured on stiff gels. Red box: apoptotic cells with high fluorescence in the Annexin V channel and low fluorescence in the DAPI channel. (B) A representative confocal image of a cell stained positively for Annexin V (red) overlaid with transmitted light DIC on stiff gels. Positively-stained cells were observed to exhibit a rounded morphology. Blue: Nucleus. Scale bar: 20 µm. (C) Quantification of apoptosis based on flow cytometry as shown in (A). Low levels of apoptosis were detected for VICs cultured on either gels or plastic plates. VICs treated with camptothecin, an apoptosis-inducing reagent, showed a much higher level of apoptosis than any gel-based culture condition or plastic plate. * indicates p<0.05.

To investigate apoptosis as a potential mechanism of the reduced proportion of myofibroblasts on stiff-to-soft gels, expression of the early apoptotic marker phosphatidylserine on the outer plasma membrane was examined. Annexin V, which binds phosphatidylserine, and DAPI staining coupled with flow cytometry was used to quantify the percentage of apoptotic cells. Live cells at an early stage of apoptosis stain positive for Annexin V and minimally for DAPI (Fig. 3A, red box). Fig. 3A shows a representative scatter plot of apoptosis staining for VICs cultured on stiff gels harvested on day 3. During 5 days of VIC culture, no significant change in cell number was observed, and on average, minimal dead cells were detected across all culture conditions based on low DAPI staining (Fig. 3A as an example). On day 3, similar percentages of apoptotic cells in VIC culture on stiff gels (3.58
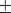
0.80%) and on soft gels (4.41
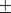
0.09%) were detected. Interestingly on day 5, we observed a slight but significant increase in apoptosis on stiff-to-soft gels (5.11
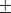
0.62%) as compared to stiff gels (2.97
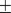
0.67%) (Fig. 3C). However, the level of apoptosis was statistically the same between VICs on soft gels (5.22
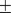
1.90%) and those on stiff-to-soft gels on day 5. As a positive control, VICs cultured on plastic plates were treated with camptothecin, an apoptosis-inducing reagent. The percentage of apoptotic cells increased from 3.72% to 32.10% (Fig. 3C) with camptothecin treatment. Additionally, we examined the morphology of the Annexin V stained cells to confirm our observations. Cells that stained positive for Annexin V expressed a rounded morphology, whereas those that did not express Annexin V maintained a spindle-shaped morphology (Fig. 3B).

### Valvular Myofibroblasts Transform into a Less Proliferative Fibroblast Phenotype on Softer Substrate

**Figure 4:**
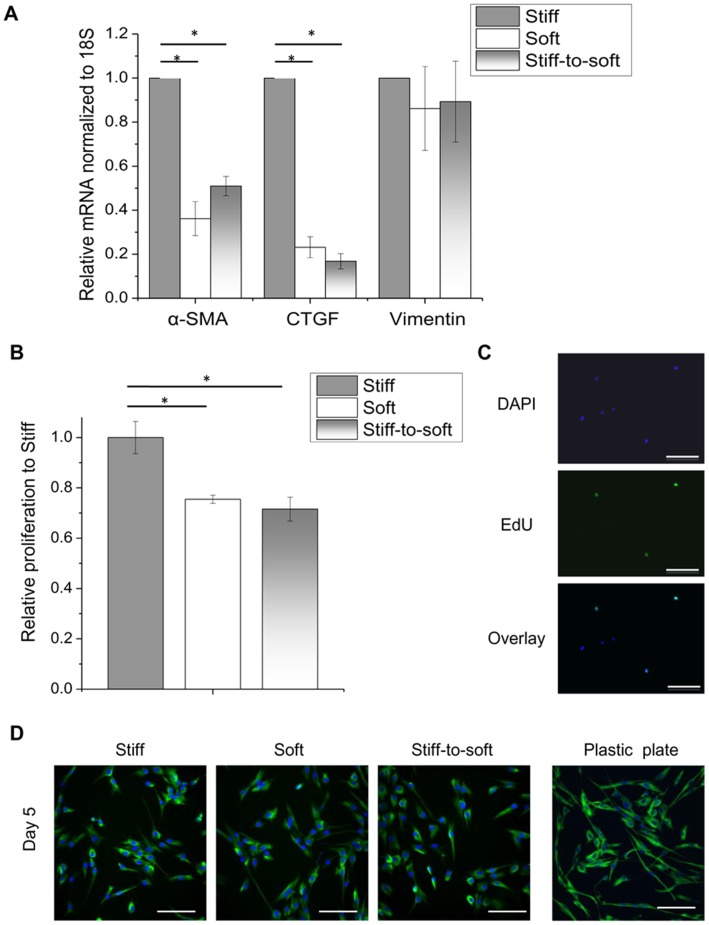
VICs switch to a less activated and less proliferative fibroblast phenotype on softer substrates. (A) 6 hours after switching stiff gels to soft on day 3, VICs were collected for mRNA quantification based on real-time PCR. Myofibroblast gene markers, α-SMA and connective tissue growth factor (CTGF), were significantly down-regulated in soft and stiff-to-soft conditions, compared with stiff. The fibroblast gene marker, vimentin, was expressed at a similar level on different substrates. (B) To measure proliferation, VICs cultured on stiff, soft or stiff-to-soft gels were chased with EdU for 3 hours on day 5. EdU incorporation into DNA was detected by labeling EdU with Alexa Fluor 488 and was quantified via flow cytometry. Relative proliferation on day 5 of VIC culture was calculated based on normalizing the percent of EdU+ cells in each condition to that of the stiff condition. VICs were less proliferative on soft and stiff-to-soft gels than on stiff gels. (C) Representative EdU staining of VICs cultured on PD-PEG gels. As expected, EdU staining (green) is co-localized with nuclei (blue). Scale bar: 100 µm. * indicates p<0.05. (D) VICs cultured on plastic plate, stiff, soft or stiff-to-soft gels were fixed on day 5 and stained for vimentin. De-activated fibroblasts on stiff-to-soft gels maintain the mesenchymal fibroblast fate. Green: vimentin. Blue: nuclei. Scale bar: 100 µm.

We hypothesized that, when the modulus of the microenvironment decreases, valvular myofibroblasts will revert to a quiescent fibroblast state. Expression of myofibroblast gene markers, including α-SMA [20], [44] and CTGF [45], and a fibroblast gene marker, vimentin [46], were measured by qRT-PCR at 6 hours after softening substrates on day 3. Fig. 4A shows that VICs on stiff-to-soft gels expressed 49% less α-SMA mRNA and 83% less CTGF mRNA than those cultured on stiff gels. VICs cultured continuously on a soft substrate expressed a similarly low level of α-SMA and CTGF as those cultured on stiff-to-soft gels. However, the expression of vimentin mRNA was not changed significantly for cells cultured on any substrates (Fig. 4A). Consistently, cells on gels with different moduli have the characteristic staining for vimentin, similar to those fibroblasts cultured on plastic plates (Fig. 4D). Next, we examined the proliferative ability of VICs grown on PD-PEG gels by quantifying the incorporation of EdU, an analogue of thymidine, during DNA synthesis. One hour after irradiating stiff gels to soften them on day 3, VICs were treated with medium containing 5% FBS for all gel conditions. This was done to induce measurable proliferation with EdU treatment for 3 hours on day 5 followed by flow cytometry. As shown in Fig. 4B, the percent of EdU+ cells on stiff-to-soft gels and soft gels was ∼30% less than those cultured on stiff gels. We also observed that more cells stalled in the G2 or mitosis (M) phase of the cell cycle on soft or stiff-to-soft gels than on stiff gels (Fig. S2). When VICs were cultured on a plastic tissue culture plate and assayed for proliferation using the same experimental conditions, there were 2 fold more EdU+ cells on the plastic tissue culture plate than on the stiff gels (data not shown). Confocal fluorescence images of samples prepared for flow cytometry confirmed that EdU staining was present in the nuclei of cells (Fig. 4C).

### Deactivated Fibroblasts on Stiff-to-soft Gels Maintain Responsiveness to a Proliferative Stimulus or TGF-β1

**Figure 5:**
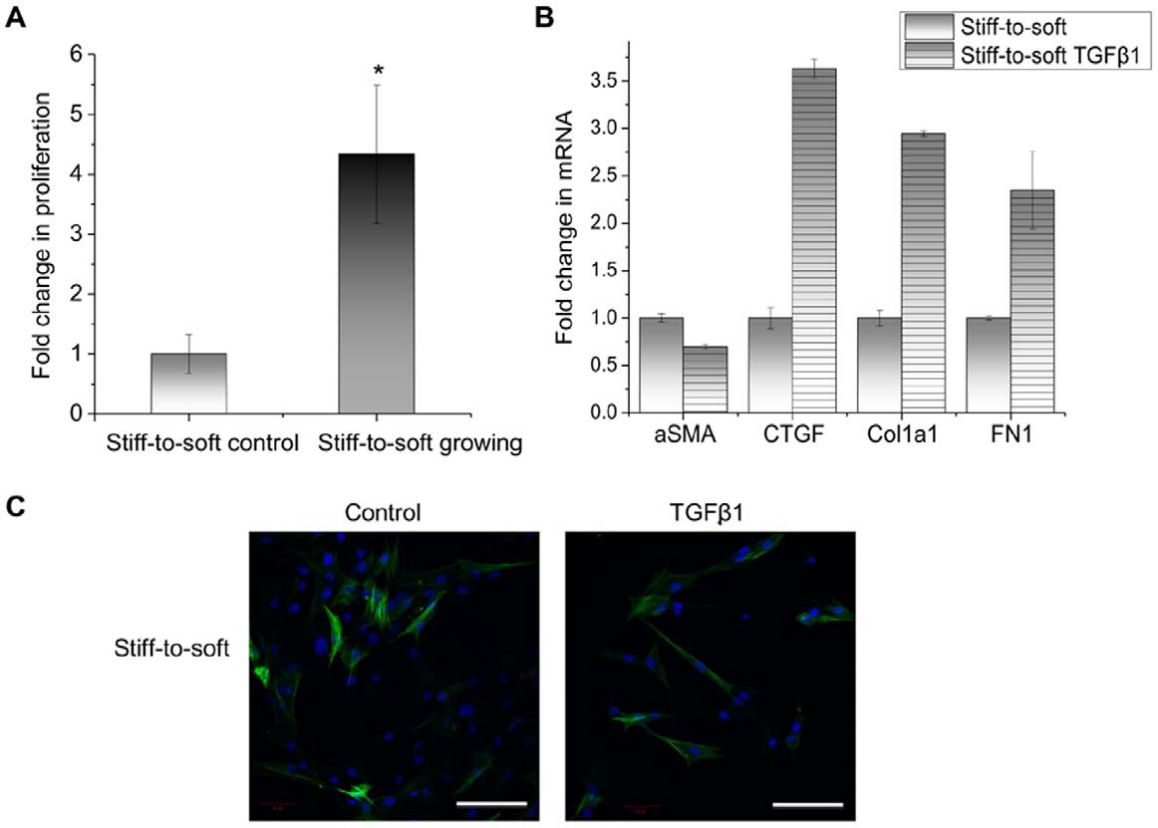
Deactivated VICs on stiff-to-soft gels enter the cell cycle with proliferative stimulus and activate myofibroblast the gene program in response to TGF-β1. VICs cultured on stiff-to-soft gels were treated with either proliferative media with 15% FBS and fibroblast growth factor 2 (FGF-2) or fibrogenic chemokine (TGF-β1) on day 4 for 24 hours. (A) Proliferation was measured by EdU chase for 1 hour on day 5. VICs treated with growth stimulus had ∼4 fold more proliferating cells than those in control medium. (B) Myofibroblast gene markers, including CTGF, collagen 1a1 (Col1a1) and fibronectin 1 (FN1), were significantly up-regulated in deactivated VICs treated with TGF-β1. The mRNA level of α-SMA was not changed significantly by TGF-β1 treatment. (C) Immunocytochemistry of α-SMA showed similar levels of myofibroblasts on stiff-to-soft gels treated with or without TGF-β1. Green: α-SMA. Blue: nuclei. These results show that the de-activated fibroblasts have the potential to proliferate and to activate fibrogenic associated genes in response to chemical cues, but a stiffer substratum is likely required for α-SMA stress fiber formation. Scale bar: 100 µm. * indicates p<0.05.

To assess whether the de-activated myofibroblast phenotype was reversible, the ability of deactivated cells to proliferate and re-activate in response to chemical cues was examined. VICs cultured on stiff-to-soft gels were treated for 24 hours with *(i)* fibroblast growth factor-2 (FGF-2) and 15% FBS to induce proliferation, or *(ii)* TGF-β1 to induce myofibroblast differentiation. As shown in Fig. 5A, deactivated VICs responded to growth stimuli and exhibited increased proliferation by ∼4 fold. These cells also exhibited up-regulated myofibroblast gene markers in response to TGF-β1 (Fig. 5B). CTGF mRNA expression was increased by 3.6 fold (Fig. 5B), and ECM genes, such as collagen 1A1 (Col1A1) and fibronectin 1 (FN1), were also significantly up-regulated, by 2.9 fold and 2.3 fold respectively, with TGF-β1 treatment (Fig. 5B). Interestingly, α-SMA mRNA level was not significantly changed, and the number of mature myofibroblasts with α-SMA stress fibers was not increased on stiff-to-soft gels with TGF-β1 treatment, (Fig. 5C). Similarly, cells cultured continuously on soft gels did not activate to α-SMA+ myofibroblasts in response to TGF-β1 (Fig. S3).

## Discussion

Here, we have begun to explore how substrate modulus serves as a mechanical cue to regulate the fate of activated valvular myofibroblasts. Our studies revealed striking phenotypic changes from activated myofibroblasts to less proliferative, quiescent-like fibroblasts when the culture substrate’s elastic modulus was reduced from 32 kPa to 7 kPa. Apoptosis was minimally associated with this de-differentiation process. Within 6 hours of *in situ* substrate elasticity reduction, gene signatures of myofibroblasts (α-SMA and CTGF) were down-regulated, while a fibroblast gene (vimentin) stayed at a similar level, confirming myofibroblast de-activation and suggesting potential signaling cascade mechanisms. Mechanically-reprogrammed VICs on stiff-to-soft gels were able to proliferate and re-initiate expression of myofibroblast genes in response to chemical cues. Considering the extensive health effects of tissue fibrosis, our study provides insight into possibly reducing fibrosis through preventing myofibroblastic activation and will assist with strategic *in vitro* tissue engineering to replace or re-organize severely fibrotic or calcified tissue.

Human tissues have stiffnesses ranging from ∼0.1 kPa to ∼20 GPa [4], [47]. To recapitulate native stiffness *in vitro*, it is critical to culture cells on substrata with a physiologically relevant stiffness for understanding their functions. Previous studies indicated that normal valve fibrosa have a bulk elastic modulus from 0.8–8 kPa [31]. When healthy valves become stenotic, osteoid, which is crosslinked collagen matrix as precursor to bone, has been detected in the valve [36]. While the stiffness of calcified valves has not been measured to our knowledge, Engler *et al.* have shown that osteoid matrix secreted by human mesenchymal stem cells has *E* ∼27
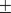
10 kPa 4. To mimic these microenvironments, we synthesized hydrogels with either a normal mesenchyme-like modulus (∼7 kPa, soft gels) or a pathological osteoid-like modulus (∼32 kPa, stiff gels) to probe the cell fate of VICs. Kloxin *et al.* previously demonstrated that valvular myofibroblast differentiation was promoted on stiff gels, but inhibited on soft gels [10]. Similarly, fibroblasts isolated from different tissues, including lung [11] and liver [9], have been shown to activate with *E* >15 kPa and maintain the α-SMA negative fibroblast phenotype when the microenvironment had *E
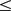
*10 kPa. We observed consistent results for VICs cultured on either stiff or soft gels (Fig. 2A). In addition, when we irradiated stiff gels with UV light to reduce the substrate modulus, valvular myofibroblasts were de-activated and lost previously formed α-SMA stress fibers (Fig. 2A). Similar behavior was observed for rat pulmonary fibroblasts (Fig. S4 and Text S1), indicating a general role of substrate modulus in regulating the differentiation of myofibroblasts.

In these experiments, VICs were cultured on a 2-dimensional surface. While this is different from the 3-dimensional (3D) valve tissue in which the endogenous cells reside, a 2D culture approach has several advantages in understanding basic biological systems. 2D surfaces of functionalized biomaterials have served as unique tools for understanding how cells collectively migrate and how they differentiate in response to stiffness or shape [4], [48], [49]. Additionally, 2D culture enables one to readily monitor and image cells over time using real time microscopy tracking tools and to collect intracellular proteins or RNA more easily compared with 3D cultures. Currently, two types of scaffolds have been used for the 3D culture of VICs, enzymatically degradable synthetic gels [50] and natural matrices comprised of collagen [44] or hyaluronic acid [51]. Both of these materials have the complication that cells are changing their mechano-environment by degrading the matrix, so it becomes difficult to know the mechanical properties of the matrix in the pericellular region. In contrast, if VICs are encapsulated in non-degradable matrices with precisely defined mechanical properties, they remain in a rounded and un-natural morphology. Thus, it is difficult to de-couple the effect of modulus and cell spreading on the cell fate within a 3D highly cross-linked matrix. For these reasons, our studies focus on isolating and understanding the effect of modulus on cell fate in 2D and believe that this knowledge will be helpful in better understanding VIC function in more complex, 3D matrices in future studies.

The fate of myofibroblasts after normal tissue repair has been an ongoing debate. In granulation and scar tissue, massive apoptosis has been observed [52]. Induction of apoptosis has been associated with matrix tension. For example, sudden release of collagen gels from their anchor causes programmed cell death in human dermal fibroblasts [53]. Additionally, compliant substrata with E <1 kPa have been shown to induce significantly higher caspase 3 activity than stiff substrata in lung fibroblasts [11]. Consistently, we observed a small but significant increase of apoptosis on stiff-to-soft gels in comparison to stiff gels on day 5 (Fig. 3C), indicating that some valvular myofibroblasts underwent apoptosis in response to reduction in modulus. However, this level of apoptosis on stiff-to-soft gels was similar to that observed for cells cultured on statically soft substrates. Additionally, the average level of apoptosis in VICs was ∼5% on stiff-to-soft gels, which was too small to account for a nearly 35% decrease in the myofibroblast population. Therefore, most myofibroblasts did not undergo apoptosis in response to substrate modulus reduction. These findings within the context of the literature suggest that there may be different thresholds of substrate modulus for regulating myofibroblast activation and apoptosis. While *E* ∼7 kPa is sufficient to de-activate valvular myofibroblasts without inducing significant apoptosis, we speculate that further reduction of *E* below or around 1 kPa would induce most cells to undergo apoptosis. Additionally, softening the substrate did not select for specific populations of cells, as the cell number counted as described in the Text S1 was not changed significantly from day 3 to day 5 across all gel moduli (Fig. S1), and cells did not proliferate or undergo apoptosis significantly over time. There were slightly fewer cells attached on soft gels at day 1 than stiff gels, so we observed fewer cells on soft gels than on stiff gels from day 3 to day 5 (Fig. S1).

Since valvular myofibroblasts did not undergo significant programmed cell death, we hypothesized that these cells de-differentiated into a dormant, or quiescent-like, fibroblast state. Myofibroblasts are differentiated from fibroblasts through increased α-SMA expression and its organization into stress fibers, which is regulated by mechanical stress [20], [54]. When cells adhere to surfaces, traction forces are generated based on the resistance of the matrix to cellular adhesion and movement [54]. Cells on substrates with higher moduli have been shown to exert higher traction forces as measured by deformation of embedded fluorescent beads [55]. Mechanical strain generated on higher substrate moduli activate intracellular signaling through p38 MAPK, Rho kinase and focal adhesion kinase to up-regulate transcription of α-SMA and subsequently incorporation of α-SMA into stress fibers [56], [57]. Our results confirmed that α-SMA stress fibers in VICs are dependent on substrate modulus. Based on Fig. 2A, α-SMA stress fibers in VICs were disassembled after 2 days of lowering substrate elasticity. Myofibroblast activation on substrates with varying moduli was independent of their time in culture (Fig. 2B), indicating minimal influence from soluble factors in the medium. On stiff-to-soft gels, we observed a higher percentage of activated myofibroblasts (∼25%) than that on soft gels (∼10%). This indicates that not every myofibroblast can be efficiently de-activated by modulus reduction on stiff-to-soft gels.

Myofibroblasts not only differ from fibroblasts in the formation of α-SMA stress fibers, but also have a distinct gene expression profile [58], [59], [60], [61]. Through previous research, gene signatures to distinguish myofibroblasts from fibroblasts have been revealed, such as α-SMA and CTGF. α-SMA is highly regulated at the transcriptional level with multiple serum response elements and CArG motifs in the promoter region of the gene [62]. CTGF expression is involved in the pathogenesis of fibrosis for various tissues and is tightly associated with the myofibroblast phenotype [45]. Both genes are more highly expressed by myofibroblasts than fibroblasts. As shown in Fig. 4A, these myofibroblast genes, α-SMA and CTGF, were significantly down-regulated with substrate modulus reduction, and the expression level of these genes was similar on stiff-to-soft gels compared with soft gels, suggesting reversion of activated VICs to a fibroblast-like phenotype. The reduction of these mRNAs was observed 6 hours after irradiating stiff gels to make them soft, indicating that cells change their molecular phenotype quickly in response to the mechanical cues. Uniquely, in comparison to substrates fabricated with discrete stiffness, changing substrate modulus *in situ* using PD-PEG gels enabled us to track dynamic transcriptional changes during myofibroblast de-activation and further reveal the molecular mechanisms regulating this process. As CTGF has been shown to be down-regulated through the YAP/TAZ pathway on soft substrata [63], it is possible that this signaling is involved in the early phase of myofibroblast deactivation on stiff-to-soft gels. Hinz *et al.* have discovered that latent TGF-β1 from the ECM is activated by contraction of α-SMA stress fibers in myofibroblasts [64]. Given that VICs rarely form α-SMA stress fibers on soft or stiff-to-soft substrata, this result indicates a limited ability to activate TGF-β1 from their microenvironment. This mechanism may act at a later phase to reinforce the un-activated fibroblast phenotype on stiff-to-soft gels.

Another functionally significant characteristic of myofibroblasts is their high rate of proliferation. Lung fibroblasts cultured on substrates with high modulus (*E* ∼100 kPa) exhibited increased myofibroblast activation and more proliferation [11]. In fibrotic lesions, a large number of myofibroblasts, generated through cell proliferation, exacerbates the inflammatory response and collagen deposition [65]. In contrast, VICs residing in healthy compliant valve matrices are mostly quiescent [26]. We found that the number of proliferating VICs was decreased by ∼30% on stiff-to-soft gels in comparison to stiff gels. This result indicates that lowering substrate modulus inhibits cell cycle progression and directs cells to a more quiescent-like phenotype. In particular, a higher fraction of the cell population stalled in the G2 or mitosis (M) phase of the cell cycle on soft or stiff-to-soft gels than on stiff gels (Fig. S2), suggesting that mechanical tension conferred by substrate modulus is an important regulator for the G2/M phase of the cell cycle. From both Fig. 4A and 4B, reducing substrate modulus not only down-regulated myofibroblast differentiation, but also controlled the proliferative response of these cells.

The myofibroblast phenotype has been suggested to be plastic, where myofibroblasts can be inhibited through different means including TGF-β1 antagonist treatment [18] and low substrate modulus [10]. If the valvular myofibroblasts were reprogrammed to quiescent fibroblasts on stiff-to-soft gels, then these cells should maintain the fibroblast gene expression and the potential to proliferate and differentiate into myofibroblasts. Vimentin is an intermediate filament protein expressed in mesenchymal cells, including fibroblasts [66]. VICs expanded on plastic plates are all positive for vimentin staining (Fig. 4D). This fibroblast property was preserved when substrate modulus was decreased. Based on Fig. 4A and 4D, mRNA and protein expression of vimentin was present at a similar level in the de-activated cells on stiff-to-soft gels, compared with cells on either stiff or soft gels, indicating that the de-activated cells were still fibroblasts. Our results also suggest that these deactivated cells are in a reversible state and respond to FGF2 and increased serum by entering the cell cycle and respond to TGF-β1 by expressing myofibroblast gene markers (Fig. 5A and 5B). Cell plasticity has become a blooming field of research with the paradigm-shifting discovery of reprogramming adult somatic fibroblasts into pluripotent stem cells by activating four transcription factors [67], [68]. A culture substratum with appropriate elastic modulus and binding epitopes shows promise as a complementary approach to reprogram the cells into a developmental stage of interest and to dynamically dictate cell phenotype and fate in a non-invasive manner.

The timing and duration of matrix signaling events are emerging as important factors in myofibroblastic differentiation plasticity and ultimate cell fate. We observe myofibroblastic de-activation of VICs with substrate modulus changes at short culture times. In complementary studies, Balestrini *et al.* have observed that lung myofibroblasts “memorized” the stiff or soft substrates on which they were propagated for 3 weeks and stayed activated or un-activated even after they had been transferred to substrates with opposite stiffness [69]. To compare, our cells have been cultured 7 days on stiff plastic plates before seeding on soft hydrogels for subsequent modulus tuning of 6 days in culture. Further, we observed similar levels of activation for freshly isolated VICs on stiff gels and stiff-to-soft gels as VICs at passage 3 (Fig. S5). This indicates that our culture of VICs on plastic plates for about a week did not change the cellular response to substrate modulus. While there could be inherent differences between valvular fibroblasts and lung fibroblasts, our results and Balestrini et al. collectively indicate that as the myofibrobalsts mature over time, there may be a time limit on their ability to revert back to fibroblasts.

Differentiation of myofibroblasts is regulated by multiple factors, including cell-cell contact [6], adhesive epitopes [70], TGF-β1 [18], [21], [71], and substrate elasticity [10], [32]. However, cells *in vivo* encounter numerous signals and integrate different types and magnitudes of signals in choosing their fate. For example, cell-cell contact prevents TGF-β1 from inducing epithelial-to-myofibroblast differentiation [72]. The ECM protein fibronectin with ED-A domain is required for TGF-β1 mediated myofibroblast differentiation [73]. The Wells group has found that portal fibroblasts need both a stiff substrate and TGF-β1 to become myofibroblasts [9]. Similarly, we observed that the myofibroblastic differentiation of VICs is regulated by both substrate stiffness and TGF-β1. When VICs were cultured on stiff-to-soft substrata (*E*, from ∼32 kPa to ∼7 kPa), they can still activate fibrogenic genes (CTGF, FN1 and Col1A1) in response to TGF-β1 (Fig. 5B). However, these cells fail to develop α-SMA stress fibers on the soft substrata even with TGF-β1 treatment (Fig. 5B and Fig. S3). We speculate that a stiffer substrate is required for mature valvular myofibroblast formation and the de-activated VICs on stiff-to-soft gels were likely in a proto-myofibroblast state when treated with TGF-β1 [20]. The PD-PEG gel system provides a powerful tool in studying cellular responses to competing signals *in vitro*, for example reduced substrate modulus while simultaneously increasing pro-fibrotic cytokines. This may provide insight into how microenvironment modulus in combination with other chemical or biological cues directs cell fate.

### Conclusion

In summary, valvular myofibroblasts were reprogrammed to fibroblast-like cells when substrate modulus was reduced with light *in situ* from *E* ∼32 kPa to *E* ∼7 kPa. This de-differentiation process is characterized by low occurrences of apoptosis, dissolution of α-SMA stress fibers, down-regulation of differentiation associated genes (α-SMA and CTGF), and a decline in cell proliferation. The de-activated fibroblasts on stiff-to-soft gels can be re-activated by FGF2 and serum to enter the cell cycle and by TGF-β1 to express fibrogenic genes, such as CTGF, Col1A1 and FN1. Our data suggest that the fate of valvular myofibroblasts is regulated by substrate elasticity independent of soluble factors. This can potentially be applied to equivalent myofibroblasts from other tissues and presents a promising approach in tempering tissue fibrosis by de-differentiating activated myofibroblasts. Our study also provides an example of dynamically reprogramming differentiated cells through substrate modulus reduction and shapes the conception of designing user-defined 2-dimensional, or even 3-dimensional, platforms for controlling the developmental stage of cells.

## Supporting Information

Figure S1 **Cell number was not changed after gel irradiation from day 3 to day 5 in culture.** Cell number was counted per field of view for both day 3 and day 5 samples. Over time, no significant change in cell number was observed for cells cultured on any of the gel moduli. There were slightly fewer cells on soft gels than on stiff gels.
(TIF)

Figure S2 **More cells reside at the G2/M phase of cell cycle on softer substrates.** VICs cultured on stiff, soft or stiff-to-soft gels were chased with EdU for 3 hours on day 5. Cell cycle profile was quantified by simultaneously labeling proliferative cells with EdU-Alexa Fluor 488 and labeling DNA content with DAPI. Fold change in percent of cells residing in the G2/M phase of the cell cycle was normalized to the stiff condition. There are 1.8 and 2.1 fold more cells in G2/M phase of the cell cycle on soft and stiff-to-soft gels respectively than those on stiff gels, indicating that substrate modulus is a regulator for cell mitosis. * indicates p<0.05.
(TIF)

Figure S3 **VICs cultured on soft gels did not respond to TGF-β1 with more α-SMA stress fiber formation.** VICs cultured on soft gels were treated with TGF-β1 on day 4 for 24 hours to induce myofibroblast differentiation. α-SMA organization was examined by immunocytochemistry. Green: α-SMA. Blue: nuclei. Few myofibroblasts with α-SMA stress fibers were observed on soft gels, and TGF-β1 did not induce further cell activation. Scale bar: 100 µm.
(TIF)

Figure S4 **Rat pulmonary myofibroblasts de-activated with reduction in substrate modulus.** Rat pulmonary myofibroblasts were stained for α-smooth muscle actin (α-SMA) after culture on stiff, soft or stiff-to-soft gels until day 5. (A) Representative staining of α-SMA to denote the myofibroblast phenotype. These cells lost α-SMA stress fibers on softer substrates. Green: α-SMA. Blue: nuclei. Scale bar: 100 µm. (B) Quantification of the percent of myofibroblasts on the substrates based on staining in (A). The percentage of myofibroblasts on stiff-to-soft gels or soft gels was significantly lower than that on stiff gels. This is consistent with the observation on valvular fibroblasts in Figure 2, indicating a general role of substrate modulus in regulating the differentiation of myofibroblasts. * indicates p<0.05.
(TIF)

Figure S5 **Freshly isolated VICs (P0 VICs) are activated on stiff gels and de-activated with reduction in substrate modulus.** Freshly isolated VICs that have not been sub-cultured on plastic plates were seeded on stiff gels. Cell activation was examined on day 5 by α-SMA immunocytochemistry. Green: α-SMA. Blue: nuclei. A similar percentage of myofibroblasts was observed for P0 VICs on stiff gels compared with P3 VICs that have been expanded on plastic plate. After gel softening with light (stiff-to-soft gel), activated P0 VICs were de-activated with significant reduction in the number of myofibroblasts. Scale bar: 100 µm.
(TIF)

Text S1 **Supplemental Materials.**
(DOC)
