# Supplementary figures and images for: Redirecting Valvular Myofibroblasts into Dormant Fibroblasts through Light-mediated Reduction in Substrate Modulus
Source: PLoS One. 2012 Jul 13;7(7):e39969. doi: 10.1371/journal.pone.0039969 (PMC3396623; doi:10.1371/journal.pone.0039969)

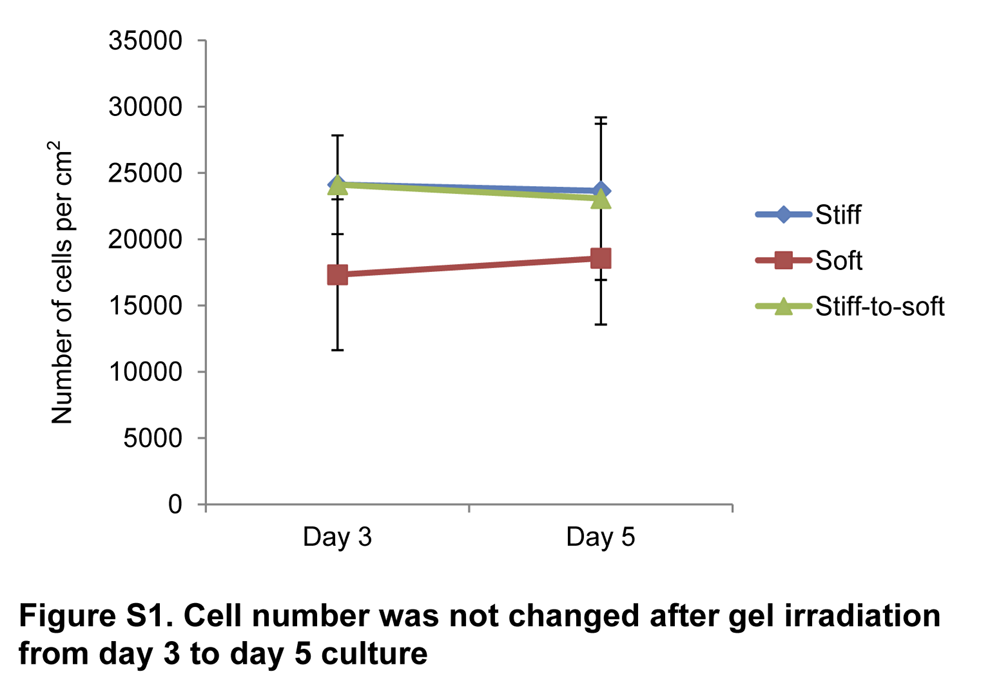

Supplement: Figure S1 — Cell number was not changed after gel irradiation from day 3 to day 5 in culture. Cell number was counted per field of view for both day 3 and day 5 samples. Over time, no significant change in cell number was observed for cells cultured on any of the gel moduli. There were slightly fewer cells on soft gels than on stiff gels. (TIF) [file pone.0039969.s001.tif]

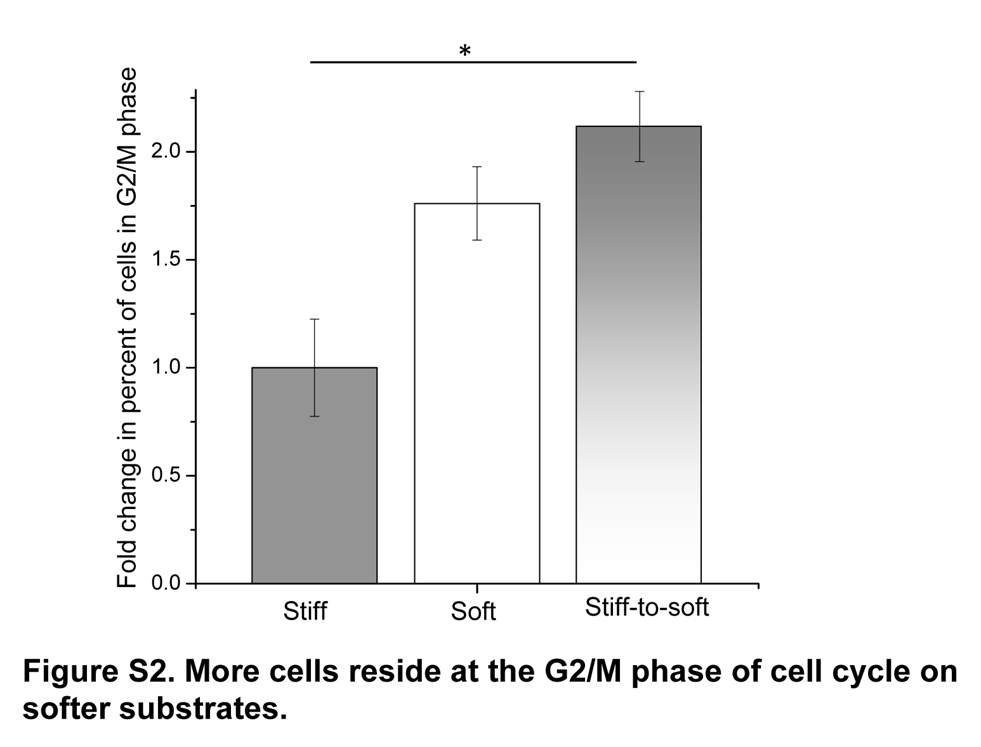

Supplement: Figure S2 — More cells reside at the G2/M phase of cell cycle on softer substrates. VICs cultured on stiff, soft or stiff-to-soft gels were chased with EdU for 3 hours on day 5. Cell cycle profile was quantified by simultaneously labeling proliferative cells with EdU-Alexa Fluor 488 and labeling DNA content with DAPI. Fold change in percent of cells residing in the G2/M phase of the cell cycle was normalized to the stiff condition. There are 1.8 and 2.1 fold more cells in G2/M phase of the cell cycle on soft and stiff-to-soft gels respectively than those on stiff gels, indicating that substrate modulus is a regulator for cell mitosis. * indicates p<0.05. (TIF) [file pone.0039969.s002.tif]

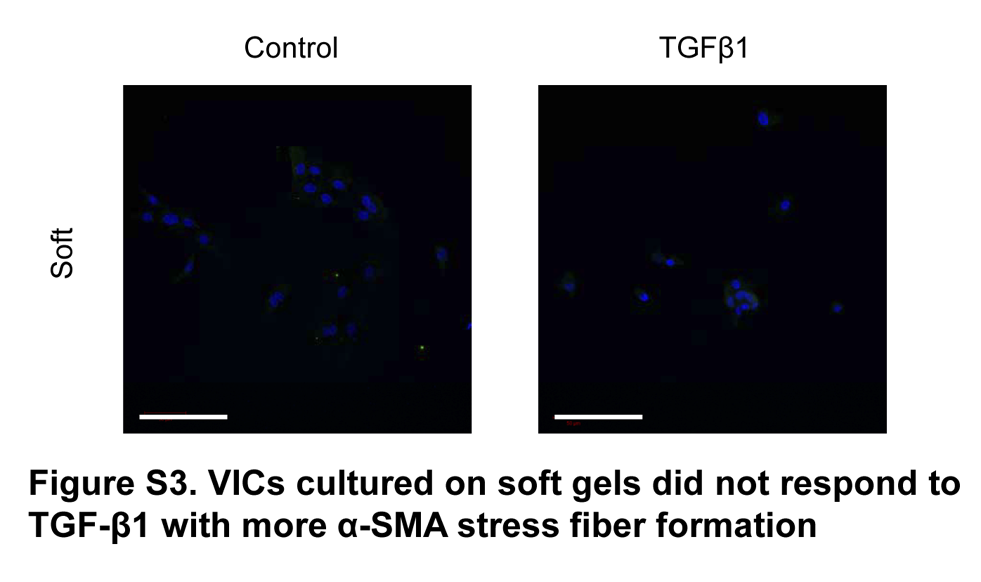

Supplement: Figure S3 — VICs cultured on soft gels did not respond to TGF-β1 with more α-SMA stress fiber formation. VICs cultured on soft gels were treated with TGF-β1 on day 4 for 24 hours to induce myofibroblast differentiation. α-SMA organization was examined by immunocytochemistry. Green: α-SMA. Blue: nuclei. Few myofibroblasts with α-SMA stress fibers were observed on soft gels, and TGF-β1 did not induce further cell activation. Scale bar: 100 µm. (TIF) [file pone.0039969.s003.tif]

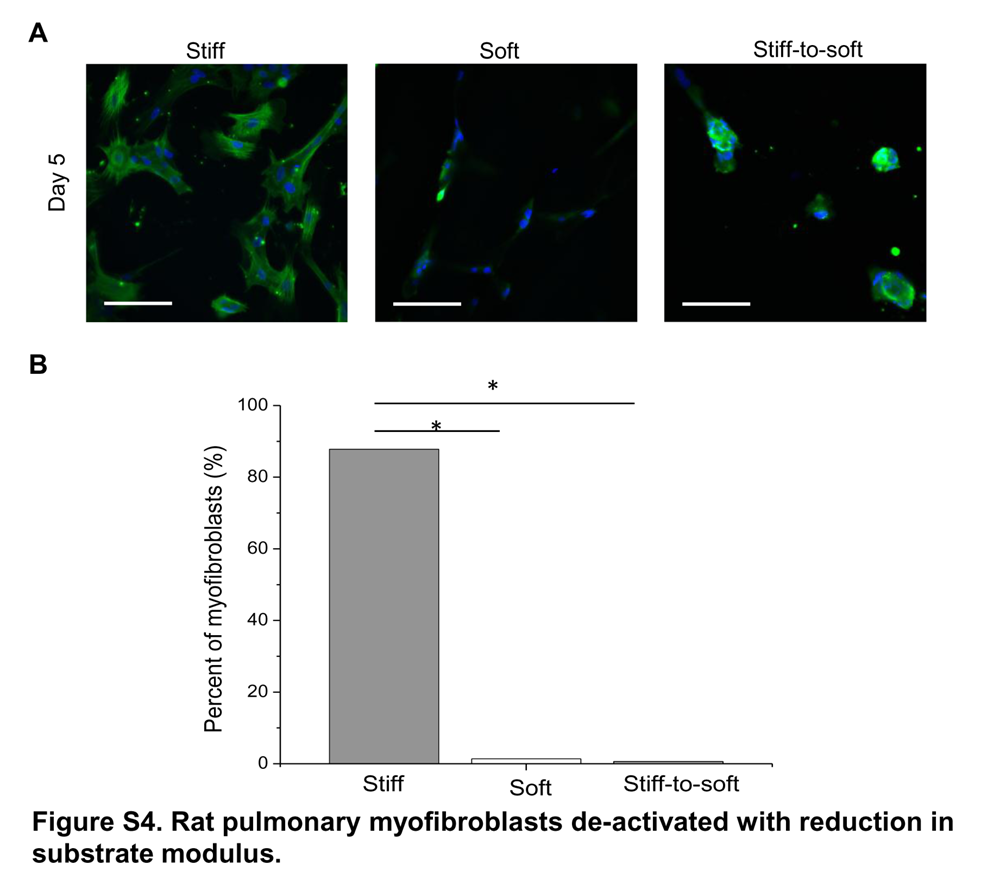

Supplement: Figure S4 — Rat pulmonary myofibroblasts de-activated with reduction in substrate modulus. Rat pulmonary myofibroblasts were stained for α-smooth muscle actin (α-SMA) after culture on stiff, soft or stiff-to-soft gels until day 5. (A) Representative staining of α-SMA to denote the myofibroblast phenotype. These cells lost α-SMA stress fibers on softer substrates. Green: α-SMA. Blue: nuclei. Scale bar: 100 µm. (B) Quantification of the percent of myofibroblasts on the substrates based on staining in (A). The percentage of myofibroblasts on stiff-to-soft gels or soft gels was significantly lower than that on stiff gels. This is consistent with the observation on valvular fibroblasts in Figure 2, indicating a general role of substrate modulus in regulating the differentiation of myofibroblasts. * indicates p<0.05. (TIF) [file pone.0039969.s004.tif]

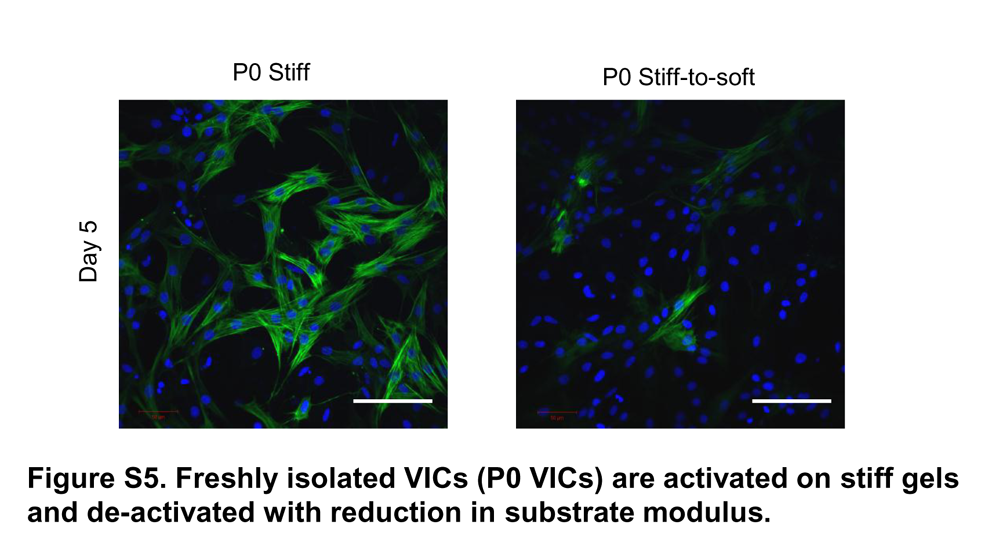

Supplement: Figure S5 — Freshly isolated VICs (P0 VICs) are activated on stiff gels and de-activated with reduction in substrate modulus. Freshly isolated VICs that have not been sub-cultured on plastic plates were seeded on stiff gels. Cell activation was examined on day 5 by α-SMA immunocytochemistry. Green: α-SMA. Blue: nuclei. A similar percentage of myofibroblasts was observed for P0 VICs on stiff gels compared with P3 VICs that have been expanded on plastic plate. After gel softening with light (stiff-to-soft gel), activated P0 VICs were de-activated with significant reduction in the number of myofibroblasts. Scale bar: 100 µm. (TIF) [file pone.0039969.s005.tif]
