# Supplementary material for: Redirecting Valvular Myofibroblasts into Dormant Fibroblasts through Light-mediated Reduction in Substrate Modulus
Source: PLoS One. 2012 Jul 13;7(7):e39969. doi: 10.1371/journal.pone.0039969 (PMC3396623; doi:10.1371/journal.pone.0039969)
Supplement: Text S1 — Supplemental Materials. (DOC) [file pone.0039969.s006.doc]

**Supplemental Material**

**Redirecting Valvular Myofibroblasts into Dormant Fibroblasts through Light-mediated Reduction in Substrate Modulus**

Huan Wang, Sarah M. Haeger, April M. Kloxin, Leslie A. Leinwand and Kristi S. Anseth^*^

* Author to whom correspondence should be addressed. kristi.anseth@colorado.edu

**This file includes:**

Supplemental Text

Supplemental Methods

Supplemental Figure captions

Supplemental Figures (5)

References

**Supplemental Text**

To explore whether substrate modulus-mediated myofibroblast de-activation is a generalized mechanism, we examined the response of another cell type, rat pulmonary fibroblasts, to *in situ* modulus reduction using the photodegradable poly(ethylene glycol) (PD-PEG) gels. Rat lung fibroblasts have been shown to activate into myofibroblasts with prominent αSMA stress fibers when cultured on tissue culture plastic plate (authors’ observation and [1]). We cultured these pulmonary fibroblasts on PD-PEG gels similarly to what was done for VICs (Fig. 1). A portion of the stiff gels were irradiated to reduce substrate modulus from ~32kPa to ~7kPa on day 3. Samples were subsequently fixed on day 5 and stained for αSMA. Rat pulmonary fibroblasts were activated to myofibroblasts on stiff gels and lost the phenotype upon modulus reduction (stiff-to-soft gels). This provides further evidence to support the general role of substrate modulus in regulating myofibroblast differentiation.

**Supplemental Methods**

**Cell count**

Samples fixed for immunostaining as described in Materials and Methods 2.4 were used for cell counting. Nuclei were stained with DAPI and imaged on an inverted epi-fluorescent microscope (Nikon) with a 20X magnification objective. The nuclei number per field of view was quantified with Image J (Analyze Particles function, NIH) with manual correction when multiple nuclei were aggregated. 6 random fields of view were counted for 3 biological replication of experiments.

**Isolation and culture of rat pulmonary fibroblasts**

Rat pulmonary fibroblasts were a gift from Dr. Leslie Leinwand’s lab (University of Colorado Boulder). Briefly, they were isolated from 6-7 week old Lewis rats, as previously described [1,2]. The cells were cultured in DMEM medium (containing 10% FBS, 50U/ml penicillin, 50μg/ml streptomycin, and 0.5µg/ml Fungizone) and seeded on PD-PEG gels at 35,000 cells/cm^2^ at passage 5. The culture was then maintained in low serum DMEM medium (1% FBS, 50U/ml penicillin, 50μg/ml streptomycin, and 0.5µg/ml fungizone) for up to 5 days. Staining for αSMA was performed as described in the “Materials and Methods 2.4 Immunocytochemistry”.

1. Rice NA, Leinwand LA (2003) Skeletal myosin heavy chain function in cultured lung myofibroblasts. The Journal of Cell Biology 163: 119-129.

2. McIntosh JC, Hagood JS, Richardson TL, Simecka JW (1994) Thy1 (+) and (-) lung fibrosis subpopulations in LEW and F344 rats. European Respiratory Journal 7: 2131-2138.
